# Genetic diversity of the conserved motifs of six bacterial leaf blight resistance genes in a set of rice landraces

**DOI:** 10.1186/1471-2156-15-82

**Published:** 2014-07-12

**Authors:** Basabdatta Das, Samik Sengupta, Manoj Prasad, Tapas Kumar Ghose

**Affiliations:** 1 Division of Plant Biology, Bose Institute, Main Campus, 93/1 A.P.C. Road, 700009 Kolkata, West Bengal, India; 2 Department of Horticulture, Institute of Agricultural Science, University of Calcutta, 35, Balligunge Circular Road, 700029 Kolkata, West Bengal, India; 3 National Institute of Plant Genome Research (NIPGR), Aruna Asaf Ali Marg, 110067 New Delhi, India

**Keywords:** Genetic diversity, BLB resistance, DNA markers, Indian landraces, Rice

## Abstract

**Background:**

Bacterial leaf blight (BLB) caused by the vascular pathogen *Xanthomonas oryzae* pv. *oryzae* (*Xoo*) is one of the most serious diseases leading to crop failure in rice growing countries. A total of 37 resistance genes against *Xoo* has been identified in rice. Of these, ten BLB resistance genes have been mapped on rice chromosomes, while 6 have been cloned, sequenced and characterized. Diversity analysis at the resistance gene level of this disease is scanty, and the landraces from West Bengal and North Eastern states of India have received little attention so far. The objective of this study was to assess the genetic diversity at conserved domains of 6 BLB resistance genes in a set of 22 rice accessions including landraces and check genotypes collected from the states of Assam, Nagaland, Mizoram and West Bengal.

**Results:**

In this study 34 pairs of primers were designed from conserved domains of 6 BLB resistance genes; *Xa1*, *xa5*, *Xa21*, *Xa21(A1)*, *Xa26* and *Xa27*. The designed primer pairs were used to generate PCR based polymorphic DNA profiles to detect and elucidate the genetic diversity of the six genes in the 22 diverse rice accessions of known disease phenotype. A total of 140 alleles were identified including 41 rare and 26 null alleles. The average polymorphism information content (PIC) value was 0.56/primer pair. The DNA profiles identified each of the rice landraces unequivocally. The amplified polymorphic DNA bands were used to calculate genetic similarity of the rice landraces in all possible pair combinations. The similarity among the rice accessions ranged from 18% to 89% and the dendrogram produced from the similarity values was divided into 2 major clusters. The conserved domains identified within the sequenced rare alleles include Leucine-Rich Repeat, BED-type zinc finger domain, sugar transferase domain and the domain of the carbohydrate esterase 4 superfamily.

**Conclusions:**

This study revealed high genetic diversity at conserved domains of six BLB resistance genes in a set of 22 rice accessions. The inclusion of more genotypes from remote ecological niches and hotspots holds promise for identification of further genetic diversity at the BLB resistance genes.

## Background

In rice more than 70 diseases caused by fungi, bacteria, viruses and nematodes are prevalent (*Oryza sativa*). The most devastating of them are the ones caused by *Magnaporthe grisea* (rice blast), *Xanthomonas oryzae* pv. *oryzae* (bacterial leaf blight, BLB) and *Rhizoctonia solani* (sheath blight). Improved agricultural practices, nutritional supplements, application of fungicides, bactericides and resistant cultivars had been used for disease control but no durable solution was available due to the breakdown of the resistance by high pathogenic variability. Hence, the search for resistant rice genotypes, particularly among the landraces, is in progress. According to Harlan [1] the extensive diverse array of rice landraces available worldwide are probable storehouses for novel alleles for many qualitative and quantitative traits. Harlan’s study emphasized that each landrace has certain unique properties or characteristics; such as early maturity, adaptation to particular soil types, resistance or tolerance to biotic and abiotic stresses, and in the end usage of the grains. India is home to many such unique landraces and the ones found in the ecological hotspots of the Indo-Burma region, and the Indian states of West Bengal, Assam, Nagaland, Mizoram and Manipur deserve special mention [2].

BLB caused by the vascular pathogen *Xanthomonas oryzae* pv. *oryzae* (*Xoo*) is one of the most serious diseases leading to crop failure in rice growing countries including Korea, Taiwan, Philippines, Indonesia, Thailand, India and China. *Xanthomonas* (from two Greek words; xanthos, meaning ‘yellow’, and monas, meaning ‘entity’) is a large genus of gram-negative and yellow-pigmented bacteria. Xoo enters rice leaf typically through the hydathodes at the leaf margin, multiplies in the intercellular spaces of the underlying epithelial tissue, and moves to the xylem vessels to cause systemic infection [3,4].

Genes conferring resistance to the major classes of plant pathogens have been isolated from a variety of plant species and are termed ‘R genes’ [5]. Comparison of the structural features and the sequences of the predicted proteins from the cloned ‘R genes’ from various plants have led to the identification of common domains which are conserved and show little variation. These conserved domains can be divided into five broad classes. They are the nucleotide-binding domain (NBD), the leucine rich repeat domain (LRR), the coiled coil domain (CC), the serine/threonine protein kinase domain and the detoxifying enzymes [5]. A total of 38 [6] BLB resistance genes (R genes) have been identified in rice, including *Xa1*, *Xa2*, *Xa3/Xa26*, *Xa4*, *xa5*, *Xa6*, *Xa7*, *xa8*, *xa9*, *Xa10*, *Xa11*, *Xa12*, *xa13*, *Xa14*, *xa15*, *Xa16*, *Xa17*, *Xa18*, *xa19*, *xa20*, *Xa21*, *Xa22(t)*, *Xa23*, *xa24(t)*, *xa25/Xa25(t)*, *Xa25*, *xa26(t)*, *Xa27*, *xa28(t)*, *Xa29(t)*, *Xa30 (t)*, *xa31(t)*, *Xa32(t)*, *xa33(t)*, *xa34(t)*, *Xa35(t)*, *Xa36(t)*. The recessive resistance genes include *xa5*, *xa8*, *xa9*, *xa13*, *xa15*, *xa19*, *xa20*, *xa24*, *xa25/Xa25(t)*, *xa26(t)*, *xa28(t)*, *xa31(t)*, *xa33(t)*, and *xa34(t)*. Of the 37, 10 BLB resistance (R) genes have been mapped on rice chromosomes 4 (*Xa1*, *Xa2*, *Xa12*, *Xa14* and *Xa25*), chromosome 5 (*xa5*), chromosome 6 (*Xa7*), chromosome 8 (*xa13*), and chromosome 11 (*Xa3*, *Xa4*, *Xa10*, *Xa21*, *Xa22*, and *Xa23*). The chromosomal locations for the rest of the BLB resistance genes still remain elusive. These R genes are known to act in a gene-for-gene manner and are the main sources for genetic improvement of rice for resistance to Xoo. Ten of the recessive R genes; *xa5*[7], *xa8*[8], *xa13*[9], *xa24*[10], *xa26, xa28*[11] and *xa32*[12] occur naturally and confer race-specific resistance. The other 3, *xa15*[13], *xa19* and *xa20*[14], were created by mutagenesis and each confers a wide spectrum of resistance to *Xoo*[11,13,15].

Six BLB resistance genes, *Xa1*, *xa5*, *Xa21*, *Xa21(A1)*, *Xa26* and *Xa27*, have been cloned, sequenced and characterized. In 1967, Sakaguchi [16] identified *Xa1* conforming a high level of specific resistance to race 1 strains of Xoo in Japan and mapped it on rice chromosome 4. The gene *xa5* is a naturally occurring mutation that is most commonly found in the Aus-Boro group of rice varieties from the Bangladesh region of Asia [7,17]. The predicted protein product of *Xa21* carries LRRs in the extracellular region and a serine/threonine kinase domain in the cytoplasm [18]. Xa21 is a member of a multigene family located on rice chromosome 11 [18,19]. Seven *Xa21* gene family members, designated A1, A2, B, C, D, E, and F, were cloned and grouped into two classes based on DNA sequence similarity [18]. Xa26 is a dominant gene coding for a LRR receptor kinase protein. It is mapped to the long arm of chromosome 11 [11,20] and was found in cultivar Mingui 63 which showed resistance against a number of *Xoo* strains both at seedling and at adult stage suggesting that it was not developmentally regulated [14]. The *Xa27* locus of rice conferred resistance to diverse strains of *Xoo,* including PXO99A, a strain isolated from rice variety IRBB27 by map-based cloning. *Xa27* is an intron-less gene and encodes a protein of 113 amino acids.

Natural selection in the ecological niches of the world has generated landraces that are highly diverse for various quality, quantity and disease resistance traits controlling loci. It is important to identify and maintain this polymorphism to widen the genetic base of the commercially cultivated varieties and to reduce pathogen pressure. According to Glaszman et al. [21] study of local sequence variation reveals the multiple examples of mutation that have taken place due to adaptation towards specific drifts and selection pressure. This adaptive neo diversity superimposes on the ancestral diversity inherited from wild relatives and forms an important section in the passport data of various accessions. It is a tedious task to put the existing natural variation to commercial use. As a step towards that process Nordborg and Weigel [22] suggested the use of genome-wide association (GWA) mapping which associates the phenotype of interest to DNA sequence variation present in an individual’s genome determined by polymorphism at various loci. GWA mapping gives much higher resolution than linkage mapping because they involve studying associations in natural populations and reflect adaptive recombination events. This kind of mapping is very useful in self fertilized species like *A. thaliana* and rice [23]. Further, in view of the challenge of assessing the diversity in large germplasm collections, the core collection concept was developed wherein diversity analysis will first be concentrated on a representative manageable sample before extending the study to a broad range of accessions [24]. Such programs have been undertaken for rice and chickpea. In accordance with such postulates the objective of this study is to analyze a small set of phenotypically variable rice accessions from BLB disease hotspot for getting a birds-eye view of the existing diversity in 6 BLB resistant gene loci of those accessions.

Reports of diversity analysis of the BLB resistance genes are available. Ullah *et al.,*[24] identified the presence of the genes *Xa4*, *xa5*, *Xa7*, and *xa13* in 52 basmati landraces and five basmati cultivars using Polymerase Chain Reaction (PCR) based methods. They also found that the gene *Xa7* was most prevalent among the cultivars and landraces while the genes *xa5* and *xa13* were confined to landraces only. Ten basmati landraces from their study had multiple resistance genes. Arif *et al.,*[25] identified the BLB resistance gene *Xa4* in 49 Pakistani rice lines. Lee *et al.,*[11] identified three rice cultivars with resistance to various Phillipino Xoo strains. The cultivar Nep Bha Bong had a new recessive gene, designated as xa26(t) for moderate resistance to races 1, 2, and 3 and resistance to race 5. The cultivar Arai Raj had a dominant gene designated as *Xa27(t)* for resistance to race 2. The cultivar Lota Sail had a recessive gene designated as *xa28(t)* for resistance to race 2. Bimolata [26] analyzed the sequence variation in the functionally important domains of *Xa27* across the *Oryza* species and found synonymous and non-synonymous mutations in addition to a number of InDels in non-coding regions of the gene. To the best of our knowledge, there is no report available on diversity of BLB resistance loci of rice landraces from the Indian states of Assam, Arunachal Pradesh, Nagaland, Mizoram, Manipur, Tripura and West Bengal.

In this study 34 pairs of primers were designed from conserved domains of the six BLB resistance genes; *Xa1*, *Xa5*, Xa21, Xa21*(A1)*, *Xa26* and *Xa27*. The designed primer pairs were used to generate PCR based polymorphic DNA profiles to detect and elucidate the genetic diversity of the six genes in the 22 rice accessions collected from West Bengal and the North Eastern States of India.

## Methods

### Plant materials

A total of 22 rice genotypes, including landraces and check genotypes, were collected from rice research stations in India. The names of the accessions, source, category and disease phenotype are given in Table 1.

**Table 1:** Name of the landraces, their source, category, disease phenotype and number of accessions

**Landrace name**	**Source**	**Category**	Disease phenotype*
Bangalakshmi	ATC Fulia	WBNA	Susceptible
Bangladeshi Patnai	ATC Fulia	WBNA	Resistant
Bhasamanik	ATC Fulia	WBNA	Resistant
Chamormoni	RRS, Chinsurah	WBNA TR	Susceptible
Dudherswar	SARF, Kashipur	WBNA TR	Susceptible
Gobindobhog	RRS, Chinsurah	WBA	Susceptible
Katarihog	RRS, Chinsurah	WBA	Resistant
Pusa Basmati 1	ATC, Fulia	EB	Susceptible
Raghusail	RRS, Chinsurah	WBNA	Resistant
Talmari	RRS, Chinsurah	WBNA	Susceptible
Taraori Basmati	ATC, Fulia	TB	Susceptible
Aijung	AAU	NA ASM	Susceptible
Boro chhaiyamora	AAU	NA ASM	Susceptible
Bhu	NBPGR, Umiam	NA MZ	Susceptible
Buhrimtui	NBPGR, Umiam	NA MZ	Susceptible
IC-524502	NBPGR, Umiam	NA NG	Susceptible
IC-524526	NBPGR, Umiam	NA NG	Susceptible
Kala Boro dhan	NBPGR, Umiam	AR ASM	Susceptible
Lal Binni	AAU	AR ASM	Susceptible
Morianghou	NBPGR, Umiam	NA MN	Susceptible
IR-72	RRS, Chinsurah	HYV	Resistant
TN-1	RRS, Chinsurah	ICV	Susceptible

AR ASM – Aromatic landraces from Assam, NA MN – Non aromatic landraces from Manipur, NA MZ – Non aromatic landraces from Mizoram, NA NG – Non aromatic landraces from Nagaland, ICV – International check variety, HYV – High yielding Variety, WBNA TR – West Bengal non aromatic Table Rice, EB – Evolved Basmati, TB – Traditional Basmati, AAU – Assam Agriculture University, ATC – Agricultural Training Centre, RRS – Rice Research Station, NBPGR – National Bureau of Plant Genetic Resources, SARF – State Agricultural Research Farm, Disease Phenotype* - disease phenotype as deduced from traditional and farmer’s knowledge and as documented by Deb (2006).

### Designing primers from conserved domains of 6 BLB resistance genes

Thirty four pairs of primers were designed from publicly available sequences (NCBI) of conserved domains of 6 BLB resistance genes using the software BatchPrimer3 (http://probes.pw.usda.gov/batchprimer3). The conserved domains are: P loop, kinase 2, trans-membrane domain and LRR domain of the *Xa1* gene; TF IIA domain of the *xa5* gene; receptor kinase domain of the *Xa26* gene; the total DNA sequence of the *Xa27* gene; signal, LRR, charged and kinase domain of the *Xa21* gene; and LRR, SNAP O11 and kinase domain of the *Xa21(A1)* gene. These primer pairs were named according to the initials of the first author and the corresponding author and were numbered from BDTG1 to BDTG34. The primer pairs were designed only from the exons such that the length of the amplified products was limited to 500 to 700 base pairs. Details of the primer names, respective resistance genes, representing protein domains, original genotypes from which the resistance genes were identified, number of exons and introns, chromosomal location in base pairs (bp) of each primer pairs and the expected length of the amplification product from the original genotype in base pairs (bp) are given in Table 2.

**Table 2:** Details of the primers used

**Primer name**	**Gene**	**Protein**	**ANN temp**	**Exon no.**	**Start (bp)**	**End (bp)**	**Forward primer**	**Reverse primer**
BDTG 1	Xa1	P Loop	59.8	1	3113	3621	5 ´-ATTAATCCACGACGACCAGG – 3 ´	5 ´-GTAGCACAAGCACCTCCTCC – 3 ´
BDTG 2		Kinase 2 & 3	60	2	3602	4031	5 ´-GAGGAGGTGCTTGTGCTACAG – 3 ´	5 ´-GGCACTGGCATTACCTTGAT – 3 ´
BDTG 3		TRANS MEM	59.5	3	4681	5200	5 ´-GGTGAGGGTGCATCAAATG – 3 ´	5 ´-TTATTCCTTCGTGGCTCTGG – 3 ´
BDTG 4			59.8	3	5167	5698	5 ´-TTGGATCATGTCTCCAACCA – 3 ´	5 ´-ACTTCAGCGCTTGCATGAT – 3 ´
BDTG 5			59.8	3	5710	6587	5 ´-CATCTATCCAACCCCTTACAGC – 3 ´	5 ´-CAAGCTTGTTCATGGATTTCAA – 3 ´
BDTG 6			60.2	3	6621	8399	5 ´-TAGAACTCAGGAGGAGGCATGT – 3 ´	5 ´-TGATTGCGGAAGGATACACA – 3 ´
BDTG 7			60.2	3	8370	8940	5 ´-AGATGGAATGTGTATCCTTCCG – 3 ´	5 ´-GGAAGGATACACCTTCCATTTTC – 3 ´
BDTG 8		LRR	59.5	4	25981	26700	5 ´-GATGGCTCCTACCGCTATCA – 3 ´	5 ´-GATGTGCAAGAATGGAGCTG – 3 ´
BDTG 9			60.9	4	26662	27231	5 ´-CTCAAATTTAGTGTCTCTGCAGCTC – 3 ´	5 ´-TCCGCGATAGTTAAGCTCTAGG – 3 ´
BDTG 10			60	4	27182	27917	5 ´-TCTGCAAGCACCTCACCTC – 3 ´	5 ´-ATGCATTGGAGCGGATTG – 3 ´
BDTG 11	Xa5	TF II A	59.9	1	406048	406306	5 ´-TTCGAGCTCTACCGGAGGT – 3 ´	5 ´-AGAAACCTTGCTCTTGACTTGG – 3 ´
BDTG 12			60.2	2	411394	411535	5 ´-TGTTCTTTTCTCAGGGCCAC – 3 ´	5 ´-AGTTTGGAATCACAGGCCAC – 3 ´
BDTG 13	Xa26	RECP Kinase	59.5	1	1500	2094	5 ´-GATGCATACTCTTGCTGCCA – 3 ´	5 ´-CAAGACTGTGCAACCCCTG – 3 ´
BDTG 14			60.1	1	2043	2695	5 ´-ACCAGCTATACGGTCCAATCC – 3 ´	5 ´-GCAAGATGCAACCATGAAAGT – 3 ´
BDTG 15			59.6	1	2716	3332	5 ´-CTATTCCTGCTTCTCTTGGCA – 3 ´	5 ´-AGCCTGACGATTTTATCAAGATG – 3 ´
BDTG 16			59.6	1	3320	3956	5 ´-CATCTTGATAAAATCGTCAGGCT – 3 ´	5 ´-GGTTGCACGAAGAAGCTCAT – 3 ´
BDTG 17			59.8	1	3968	4492	5 ´-CGATGATAGCATGTTGGGC – 3 ´	5 ´-AAAAACTATTAAGTACCTGGTGCCAT– 3 ´
BDTG 18			59.9	1	4574	5141	5 ´-TGAGCAGAGTATGGGACTCTAGG – 3 ´	5 ´-ACACCAACTATAAATTGTTGCAGAAC – 3 ´
BDTG 19	Xa27		59.9	1	1518	1909	5 ´-GAAGCCACACACACTGAGACA – 3 ´	5 ´-CGGAGGAGAACTAGAGAGACCA – 3 ´
BDTG 20	Xa21	Signal	59.7	1	8	208	5 ´-CACTCCCATTATTGCTCTTCG – 3 ´	5 ´-ACACAACACCCACCCATGT – 3 ´
BDTG 21		LRR	61.8	2	260	760	5 ´-GCTCCTCCAACCTGTCCG – 3 ´	5 ´-TAAACGCTCTTAGAGACGAAAGGT – 3 ´
BDTG 22			59.7	2	723	1314	5 ´-CAATTCTATCTGGAACCTTTCGTC – 3 ´	5 ´-ACCGCTCAAGTTGTTTTCGT – 3 ´
BDTG 23			60	2	1279	1880	5 ´-GGCATTCTACTCGCCTACGA – 3 ´	5 ´-GCATTGCCTTGGATTGAGAT – 3 ´
BDTG 24		Charged	59.8	3	1913	2620	5 ´-TGCCTCGATGTTGTCCATTA – 3 ´	5 ´-TCAATGAGGTCCCATCAACA – 3 ´
BDTG 25		Kinase	60.1	4 & 5	2651	3919	5 ´-AGGGACAATTGGCTATGCAG – 3 ´	5 ´-AGAATTCAAGGCTCCCACCT – 3 ´
BDTG 26	Xa21(A1)	LRR	59.8	1	4802	5082	5 ´-TGTTGTTCTCTGCGCTGC – 3 ´	5 ´-CGTCCTGAGGAAGGATAGGTT – 3 ´
BDTG 27			59.6	1	5051	5459	5 ´-CATCGCTGGGCAACCTAT – 3 ´	5 ´-TTGGACACGACTTCAAATATGG – 3 ´
BDTG 28			59.6	1	5406	5803	5 ´-CCCAGATCCTATTTGGAACATC – 3 ´	5 ´-TGGAAACAGAATCAGGGAGG – 3 ´
BDTG 29			59.9	1	5763	6173	5 ´-AGGTTGCAAATTTGGTGGAG – 3 ´	5 ´-GGAATGCTAAATATTTCAATGGGA – 3 ´
BDTG 30			60.2	1	6140	6531	5 ´-TAGGGCAAATTCCCATTGAA – 3 ´	5 ´-AAAACACCATTGGTTGGCA – 3 ´
BDTG 31			59.9	1	6484	6889	5 ´-CTTTCGTTCAACAGCTTCCAC – 3 ´	5 ´-CACCATCTTGACTATCAAATTCTCC – 3 ´
BDTG 32			59.9	1	6859	7422	5 ´-CTTTCGTTCAACAGCTTCCAC – 3 ´	5 ´-CAATGAAAGGAGGTAGACATAAACAGT – 3 ´
BDTG 33		SNAP	60.2	2	7395	7610	5 ´-ACTGTTTATGTCTACCTCCTTTCATTG – 3 ´	5 ´-AATAGATTTGCTACGGTCGAACA – 3 ´
BDTG 34		Kinase	59.7	3	7718	8081	5 ´-TTTGTTATGGAATTCTAGTGTTGGAA – 3 ´	5 ´-CCAACATAACATCAGCATGTCTC – 3 ´

Gene - Resistance genes from which the primers were designed; Protein - Protein coded by the DNA sequence amplified by the corresponding primer; Ann Temp – Annealing Temperature of the respective primer pair; Exon no. - Exon of the original gene from which primer pair was designed; Start – expected start point of the amplification product with respect to the original gene sequence, End – Expected end point of the amplification product with respect to the original gene sequence.

### Isolation of rice genomic DNA and PCR amplification

Total genomic DNA was isolated from ten 3 day-old rice seedlings using the method of Walbot [27] with modifications. The DNA was PCR amplified using a protocol standardized in our lab and used in our previous paper [28].

### Polyacrylamide gel electrophoresis and allele scoring

The PCR products were resolved in 6% polyacrylamide gel using the procedure described by Sambrook et al. [29]. The gel staining, visualization and assignment of alleles were done according to protocols in our previous paper [28]. Null alleles were assigned when no amplification product was generated [30]. When an allele was found in less than 5% of the germplasms under study, it was designated as rare [31].

### Calculation of polymorphism information content (PIC) value

The polymorphism information content (PIC) value for the primer pairs was calculated using the formula given by Anderson *et al.*[32] for self pollinated species

$${\text{PIC}}_{i}=\;1\;–{\text{∑}}_{i=1}^{n}{P^{2}}_{\mathrm{ij}},$$

where Pij is the frequency of the j^th^ allele for the i^th^ marker.

### Genetic diversity analysis using PCR amplification profiles

A genetic similarity matrix between all possible combinations of pairs of rice accessions was made using Jaccard’s co-efficient [33] and the NTSYS-pc software package, version 2.02e, [34]. This similarity matrix was used to make a phylogenetic tree using the Unweighted Pair-Group Method of Arithmetic average (UPGMA) and Neighbor-Joining (NJoin) module of the NTSYS-pc. Support for clusters was evaluated by bootstrap analysis using WinBoot software [35] through generating 1,000 samples by re-sampling with replacement of characters within the combined 1/0 data matrix.

### Sequencing and analysis of rare alleles

The DNA was eluted from the bands of rare alleles using QIAquick Gel Extraction Kit following manufacturer’s protocol. The eluted DNA was sequenced through outsourcing and the sequences were submitted to NCBI. For finding the homology and conserved domains, the sequences were BLAST [36] searched against the non-redundant database of NCBI using default parameters. Apart from NCBI BLAST, homology search for the obtained sequences were done using the “blastn” option of the Rice Annotation Database (http://rice.plantbiology.msu.edu).

## Results

### Analysis of PCR profiles

The summary of the data of the PCR profiles of the 22 accessions using the 34 pairs of primers is given in Table 3. All the 34 primer pairs produced polymorphic profiles and a total of 140 alleles were identified including 41 rare alleles. There were no unique alleles detected. The number of alleles ranged from 2 to 8 with an average of 4.06 alleles/primer pair. The primer pairs amplifying various regions of the LRR domain (Table 2) on an average produced 4.6 alleles/primer pair. Primer pairs amplifying the regions of kinase domain on an average produced 3.8 alleles/primer pair.

**Table 3:** Maximum and minimum band length, number of alleles (T), null (N) and rare (R) alleles with names of genotypes and PIC values for each primer pair

**Marker name**	**Mol. wt. min.**	**Mol. wt. max.**	**PIC value**	**Number of alleles**				**R**	**N**
				**T**	**WB**	**NE**	**C**		
BDTG1	295	310	0.17	2	1	2	1	0	0
BDTG2	301	405	0.43	4	4	2	1	1	2
BDTG3	495	511	0.43	2	2	2	1	0	2
BDTG4	331	335	0.30	2	2	2	1	0	0
BDTG5	395	405	0.32	4	2	3	1	0	0
BDTG6	485	505	0.24	2	1	2	1	0	0
BDTG7	495	505	0.40	2	2	2	1	0	0
BDTG8	490	500	0.35	2	2	1	2	0	0
BDTG9	400	410	0.62	4	4	3	1	0	3
BDTG10	490	893	0.71	5	4	3	1	2	3
BDTG11	158	285	0.61	4	4	2	1	1	1
BDTG12	256	766	0.50	5	5	3	2	2	1
BDTG13	495	968	0.43	4	4	2	1	1	0
BDTG14	480	490	0.61	4	2	4	1	0	0
BDTG15	490	500	0.58	2	2	2	2	0	0
BDTG16	485	500	0.50	2	2	2	1	0	0
BDTG17	485	500	0.50	2	2	2	1	0	0
BDTG18	339	395	0.79	8	3	4	2	4	1
BDTG19	230	240	0.70	6	4	5	2	3	2
BDTG20	180	210	0.73	7	5	3	2	2	0
BDTG21	441	530	0.76	7	5	5	2	2	1
BDTG22	492	561	0.63	4	3	2	1	2	2
BDTG23	490	578	0.78	7	7	5	2	0	0
BDTG24	510	678	0.72	5	5	4	1	1	0
BDTG25	170	185	0.72	4	3	3	1	1	0
BDTG26	248	515	0.79	8	4	6	2	4	0
BDTG27	335	451	0.73	4	4	4	1	2	2
BDTG28	359	415	0.62	3	2	3	1	1	2
BDTG29	345	387	0.79	8	3	6	1	5	1
BDTG30	410	420	0.66	5	2	3	2	2	3
BDTG31	282	384	0.48	2	1	2	1	2	0
BDTG32	490	503	0.30	2	2	2	1	0	0
BDTG33	210	267	0.58	4	4	2	1	1	0
BDTG34	279	511	0.58	4	4	2	1	2	0
			18.93	140	106	100	44	41	26
			0.56	4.12	3.12	2.94	1.29	1.21	0.76

Mol. wt. min – minimum molecular weight obtained from the alleles of the concerned primer; Mol. wt. max - maximum molecular weight obtained from the alleles of the concerned primer; WB – West Bengal; NE – North Eastern States; C – Check accessions.

The PIC value ranged from 0.16 for the least informative primer pair BDTG1 to 0.79 for the most informative primer pairs BDTG18, BDTG26 and BDTG29. The average PIC value was 0.56/primer pair.

### Diversity in the six loci in this set of rice accession

The diversity generated by the 34 primer pairs in this set of rice accession is given in Additional file 1: Table S1. Briefly the highest variation was found in the locus *Xa21(A1)* between 4802 bp to 5082 bp (exon1, LRR domain, BDTG26) and between 5763 bp to 6173 bp (exon 1, LRR domain, BDTG29); and in the *Xa26* locus between 4574 bp to 5141 bp (exon1, BDTG18), producing 8 alleles each. Three regions in the locus *Xa21*, from 8 bp to 208 bp (exon 1, the Signal domain, BDTG20), from 260 bp to 760 bp (exon 2, the LRR domain, BDTG21) and from 1279 bp to 1880 bp (exon 2, the LRR domain, BDTG23) produced 7 alleles each. Although the *Xa27* locus was small, 392 bp long (1518 bp to 1909 bp), the primer pairs BDTG19 generated 6 alleles including 3 rare 2 null alleles. The next most variable region was in the *Xa1* locus between 27182 bp to 27917 bp (exon 4, LRR domain, BDTG10), which produced 5 alleles. The region of TFIIA domain from 406048 bp to 406306 bp of locus *xa5* (exon1, BDTG 11) produced 4 alleles including one rare allele and one null allele. The other most variable regions identified within the different loci are given in Additional file 1: Table S1.

### Genetic diversity within the different categories of landraces

The West Bengal accessions produced a total of 107 alleles with an average of 3.15 alleles/primer pair. In this group, the highest number of alleles was generated by the primer pair BDTG23, while only one allele each was produced by BDTG6 and BDTG31. The North Eastern accessions produced a total of 100 alleles with an average of 2.94 alleles/primer pair. While the highest number of 6 alleles was generated by BDTG29, only 1 allele each was produced by BDTG8 and BDTG26. The check varieties comprised of one resistant and one susceptible accession. Out of the 41 rare alleles, 8 were produced by the resistant West Bengal landrace Bhasamanik and 7 each were produced by the resistant landraces Raghusail and Bangladeshi Patnai. Four rare alleles were identified in the Assamese aromatic landrace Lal binni and 2 rare alleles each were identified in the landraces Aijong, IC524526, IC524502 and Gobindobhog.

### Dendrogram from the genetic similarity values

**Figure 1:**
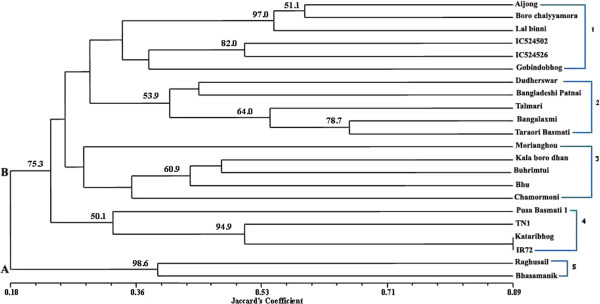
**Dendrogram showing genetic relationship among 22 rice accessions based on Jaccard's genetic similarity matrix derived from 140 alleles at 6 BLB resistance gene loci.** The major clusters are indicated on the left margin and the sub-clusters are indicated on the right margin.

In the dendrogram the similarity between the rice accessions ranged from 18% to 89% and on this basis they were divided into 2 major clusters A and B (Figure 1). Cluster A separated out at 18% level of similarity and consisted of Raghusail and Bhasamanik, both of which were resistant accessions from West Bengal. Cluster B was subdivided into 4 different sub clusters. Cluster 1 segregated out at 53% level of similarity and included the North Eastern accessions Aijong, Boro Chhaiyamora, IC524502, IC524526 and Lal Binni along with the West Bengal accession Gobindobhog. All the accessions in cluster 1 were BLB susceptible. Cluster 2 segregated at 28.8% level of similarity and included the West Bengal accessions Dudherswar, Bangladeshi Patnai, Talmari, Bangalaxmi and Taraori Basmati, all of which were susceptible. Cluster 3 separated out at 28% level of similarity and consisted of Morianghou, Kala boro dhan, Buhrimtui and Bhu from the North Eastern States along with Chamormoni – an accession from West Bengal. Cluster 4 consisted of the accessions Pusa Basmati 1, the susceptible check TN1, Kataribhog - a resistant accession from West Bengal and IR72 - a resistant check.

### Homology searches for the sequences of the rare alleles

A total of forty one rare alleles were sequenced. Of these, 40 were submitted to and were assigned accession numbers by NCBI. The accession numbers of the sequences, details of sequence homology, and the details of the conserved domains corresponding to each sequence is given in Table 4. Fifteen of the sequences were from the North Eastern accessions and 25 sequences were from the West Bengal accessions. BLAST searches using the NCBI database revealed that six rare alleles from the North East were homologous to sequences of BLB resistance genes of *Oryza sativa* japonica. Three of the rare alleles were homologous with sequences of the *Xa21* gene of *O. longistaminata*. Two rare alleles were homologous to sequences of *Xa1* and *Xa21(A1)* gene of *O. sativa* indica and one rare allele each was homologous to the *Xa21* gene sequence from *O. rufipogon* and Xa27 gene sequence of *O. officinalis* ecotype IC203740. The rare alleles from HR806765 and JM426578 from the North Eastern landraces Buhrimtui and Aijong respectively did not show any homology to the existing database.

**Table 4:** Details of the sequenced rare alleles obtained from this study and homology searches with NCBI

**Primer name**	**Gene**	**Sequenced rare allele**	**GenBank Acc No.**	**L**	**Sequence producing the most significant alignment**	**E-value**	**Name of conserved domain present**	**Domain ID**	**E-value**
BDTG2	Xa1	Raghusail	HR575926	301	*Oryza sativa* Japonica Group cDNA	2e-135	BED zinc finger	cl02703	2.74e-16
BDTG10	Xa1	Raghusail	HR575924	893	*Oryza sativa* indica mRNA for XA1	3e-124	-	-	-
		IC524526	HR806763	701	*Oryza sativa* indica mRNA for XA1	0.0	-	-	-
BDTG11	Xa5	Raghusail	HR614233	158	*Oryza sativa* Indica Group cultivar IRGC 27045 xa5 gene	4e-56	-	-	-
BDTG12	Xa5	Raghusail	HR575927	631	*Oryza sativa* Indica Group cultivar IRGC 27045 xa5 gene	2e-135	-	-	-
		Bangladeshi Patnai	HR614234	766	*Oryza sativa* Indica Group cultivar IRGC 27045 xa5 gene	2e-135	-	-	-
BDTG13	Xa26	Bhasamanik	HQ832768	968	*Oryza sativa* isolate BDTG13-Bhasa receptor kinase (Xa26) gene,	0.0	-	-	-
BDTG18	Xa26	Lal Binni	HR806757	539	*Oryza sativa* (japonica cultivar-group) bacterial blight resistance protein XA26 (Xa26) gene, complete cds	0.0	-	-	-
		Buhrimtui	HR806765	536	-		-	-	-
		Desi dhan	HR806766	532	*Oryza sativa* (japonica cultivar-group) bacterial blight resistance protein XA26 (Xa26) gene, complete cds bacterial blight resistance protein XA26 (Xa26) gene, complete cds	0.0	-	-	-
		Raghusail	HR575921	490	*Oryza rufipogon* receptor kinase-like protein, partial cds	0.0	Leucine-rich repeat receptor-like protein kinase	PLN00113	1.23e-05
BDTG 19	Xa27	Aijong	JM426578	638	-	-	-	-	-
		Morianghou	JM426580	367	*Oryza officinalis* ecotype IC203740 bacterial blight resistance protein Xa27 (Xa27) gene, complete cds	0.0	-	-	-
BDTG20	Xa21	Aijong	HR806747	542	*Oryza sativa* japonica Group Os11g0559200 mRNA	5e-65	Leucine rich repeat N-terminal domain	cl08472	1.90e-07
		Bangladeshi Patnai	HR806741	188	*Oryza sativa* Indica Group Xa21 gene for receptor kinase-like protein, complete cds, cultivar:II you 8220	9e-68	Leucine rich repeat N-terminal domain	cl08472	1.77e-06
BDTG21	Xa21	IC524526	HR806762	530	*Oryza rufipogon* Xa21F pseudogene, strain:W149	0.0	Leucine-rich repeat receptor-like protein kinase	PLN00113	2.08e-17
		Bhasamanik	HR806751	451	*Oryza rufipogon* Xa21F pseudogene, strain:W149	0.0	Leucine-rich repeat receptor-like protein kinase	PLN00113	7.76e-17
BDTG22	Xa21	Bangladeshi Patnai	HR806742	561	*Oryza rufipogon* Xa21F pseudogene, strain:W1236	0.0	Leucine-rich repeat receptor-like protein kinase	PLN00113	1.35e-21
BDTG24	Xa21	Bhasamanik	HR806749	678	*Oryza rufipogon* Xa21F pseudogene, strain:W149	0.0	Protein Kinases, catalytic domain	cl09925	6.18e-05
BDTG25	Xa21	Bangladeshi Patnai	HR806743	1 kb	*Oryza rufipogon* Xa21F pseudogene, strain:W593	0.0	-	-	-
BDTG26	Xa21(A1)	IC524502	HR806759	248	*Oryza longistaminata* receptor kinase-like protein gene, family member A1	7e-110	Leucine rich repeat N-terminal domain	cl08472	1.56e-09
		Raghusail	HR575925	268	*Oryza longistaminata* receptor kinase-like protein gene, family	2e-144			
		Aijong	HR806748	494	*Oryza sativa* Japonica Group Os11g0559200 mRNA	3e-72	-	-	-
		Lal Binni	HR806755	515	*Oryza sativa* Indica Group DNA, chromosome 8, BAC clone: K0110D12	5e-70	-	-	-
		Bhasamanik	HQ832770	457	*Oryza longistaminata* receptor kinase-like protein gene, family member A1	6e-116	-	-	-
BDTG27	Xa21(A1)	Bangladeshi Patnai	HR806744	366	*Oryza longistaminata* receptor kinase-like protein gene, family member A1	2e-78	-	-	-
		Raghusail	HR575922	325	*Oryza longistaminata* receptor kinase-like protein, family member A2	4e-104	Leucine-rich repeat receptor-like protein kinase	PLN00113	1.15e-09
BDTG28	Xa21(A1)	Bhasamanik	HR806752	359	*Oryza longistaminata* receptor kinase-like protein gene, family member A1	1e-139	Leucine-rich repeat receptor-like protein kinase	PLN00113	1.15e-09
BDTG29	Xa21(A1)	Bhasamanik	HR806750	377	*Oryza sativa* receptor kinase-like protein gene family member E	2e-146	Leucine-rich repeat, ribonuclease inhibitor (RI)-like subfamily.	PLN00113	2.67e-27
		IC524526	HR806761	379	*Oryza longistaminata* receptor kinase-like protein, complete cds and family member C,	9e-141	-	-	-
		Bangladeshi Patnai	HR806745	382	*Oryza sativa* Japonica Group Os11g0559200 mRNA,	9e-141	Leucine-rich repeat, ribonuclease inhibitor (RI)-like subfamily.	cl12243	8.45e-05
		Lal Binni	HR806761	387	*Oryza longistaminata* receptor kinase-like protein complete cds and family member C	9e-141	Leucine-rich repeat receptor-like protein kinase	cl15309	1.15e-09
		IC524502	HR806760	345	*Oryza sativa* Japonica Group Os11g0559200 mRNA	1e-134	-	PLN00113	1.79e-07
BDTG30	Xa21(A1)	Gobindobhog	HR806764	323	*Oryza sativa* Japonica Group Os11g0559200 (Os11g0559200) mRNA	2e-137	Leucine-rich repeat receptor-like protein kinase	PLN03150	3.69e-11
		Bangladeshi Patnai	HR806746	328	*Oryza sativa* Japonica Group Os11g0559200 mRNA	1e-134	Catalytic NodB homology domain of the carbohydrate esterase 4 superfamily	PLN00113	8.21e-12
BDTG31	Xa21(A1)	Bhasamanik	HR806753	376	*Oryza sativa* Japonica Group Os11g0559200 mRNA	0.0	-	-	-
		Lal Binni	HR806758	384	*Oryza sativa* Japonica Group Os11g0559200 mRNA	1e-173	-	-	-
BDTG33	Xa21(A1)	Bhasamanik	HR806754	267	*Oryza longistaminata* receptor kinase-like protein gene, family member A1	9e-30	-	-	-
BDTG34	Xa21(A1)	Raghusail	HR575923	279	*Oryza longistaminata* receptor kinase-like protein gene, family member A1	2e-110	-	-	-
		Gobindobhog	HR806767	347	*Oryza longistaminata* receptor kinase-like protein gene, family member A1	5e-153	Sugar transferase, PEP-CTERM/EpsH1 system associated;	8.96e-04	4.35e-04

GenBank Acc No. – accession number of the sequences given by GenBank, L – length of sequence in bp.

Out of the 25 rare alleles from the West Bengal landraces, Raghusail and Bhasamanik contributed 8 rare alleles each and Bangladeshi Patnai contributed 7 alleles. Eight rare alleles were homologous to sequences from *O. longistaminata* and 7 rare alleles were homologous to *O. sativa* indica sequences from the NCBI database. Five rare alleles each were homologous to sequences of *O. rufipogon* and *O. sativa* indica.

Results of homology search using the Rice Genome Annotation Project (RGAP) Database are given in Table 5. The name of the locus which produced the most significant match, description of the matched locus, E-value and details of the Pfam hits are shown in the table. According to this database most of the rare alleles were homologous to sequences of receptor kinase like proteins.

**Table 5:** Details of the sequenced rare alleles obtained from this study and homology searches with Rice annotation Database

**Primer name**	**Gene**	**Sequenced rare allele**	**GenBank Acc no.**	**L**	**Significant match with locus**	**Description of matched locus**	**E-value**	**Pfam hits**		
								**Name**	**Accession**	**E-value**
BDTG2	Xa1	Raghusail	HR575926	301	LOC_Os04g53120	NB-ARC domain containing protein, expressed	6.9e-56	zf-BED	PF02892.8	1.8e-12
BDTG10	Xa1	Raghusail	HR575924	893	LOC_Os04g53160	NBS-LRR disease resistance protein, putative, expressed	2.4e-66	zf-BED	PF02892.8	4.4e-07
		IC524526	HR806763	701	AB002266	NBS-LRR disease resistance protein, putative, expressed	6.2e-82	zf-BED	PF02892.8	4.4e-07
BDTG11	Xa5	Raghusail	HR614233	158	LOC_Os05g01710	Transcription initiation factor IIA gamma chain, putative, expressed	5.0e-24	TFIIA_gamma_N	PF02268.9	5..2e-24
BDTG12	Xa5	Raghusail	HR575927	631	LOC_Os05g01710	Transcription initiation factor IIA gamma chain, putative, expressed	1.8e-11	TFIIA_gamma_N	PF02268.9	5..3e-24
		Bangladeshi Patnai	HR614234	766	LOC_Os01g08330	Aspartic proteinase nepenthesin-1 precursor, putative, expressed	6.7e-05	Asp	PF00026.16	8.6e-25
BDTG13	Xa26	Bhasamanik	HQ832768	968	LOC_Os09g07440	Retrotransposon protein, putative, unclassified, expressed	3.4e-05	Plant_tran	PF04827.7	7.5e-10
BDTG18	Xa26	Lal Binni	HR806757	539	LOC_Os11g47000	Receptor-like protein kinase precursor, putative, expressed	1.0e-101	LRR_1	PF00560.26	0.47
		Buhrimtui	HR806765	536	LOC_Os05g26090	Transposon protein, putative, CACTA, En/Spm sub-class	0.00042	-	-	-
		Desi dhan	HR806766	532	LOC_Os11g47000	Receptor-like protein kinase precursor, putative, expressed	2.6e-101	LRR_1	PF00560.26	0.47
		Raghusail	HR575921	490	LOC_Os11g36180	Receptor kinase, putative, expressed	1.8e-86	LRR_1	PF00560.26	0.26
BDTG 19	Xa27	Aijong	JM426578	638	LOC_Os08g37540	Retrotransposon protein, putative, Ty3-gypsy subclass, expressed	0.019	Transposase_28	PF04195.5	2.6e-101
		Morianghou	JM426580	367	AY986493	Oryza sativa (indica cultivar-group) Xa27 (Xa27) mRNA, Xa27-IRBB27 allele, complete cds	2.3e-72	-	-	-
BDTG20	Xa21	Aijong	HR806747	542	LOC_Os11g35500	Receptor-like protein kinase 5 precursor, putative, expressed	3.7e-27	LRRNT_2	PF08263.5	5.9e-11
		Bangladeshi Patnai	HR806741	188	LOC_Os11g35500	Receptor-like protein kinase 5 precursor, putative, expressed	8.6e-23	LRRNT_2	PF08263.5	5.9e-11
BDTG21	Xa21	IC524526	HR806762	530	LOC_Os11g36180	Receptor kinase, putative, expressed	4.9e-52	LRRNT_2	PF08263.5	2.3e-10
		Bhasamanik	HR806751	451	LOC_Os11g36180	Receptor kinase, putative, expressed	4.9e-52	LRRNT_2	PF08263.5	2.3e-10
BDTG22	Xa21	Bangladeshi Patnai	HR806742	561	LOC_Os11g36180	Receptor kinase, putative, expressed	7.9e-115	LRRNT_2	PF08263.5	2.3e-10
BDTG24	Xa21	Bhasamanik	HR806749	678	LOC_Os11g36180	Receptor kinase, putative, expressed	3.8e-135	LRRNT_2	PF08263.5	2.3e-10
BDTG25	Xa21	Bangladeshi Patnai	HR806743	1 kb	LOC_Os11g36180	Receptor kinase, putative, expressed	6.2e-119	LRRNT_2	PF08263.5	2.3e-10
BDTG26	Xa21(A1)	IC524502	HR806759	248	LOC_Os11g35500	Receptor-like protein kinase 5 precursor, putative, expressed	3.4e-43	LRRNT_2	PF08263.5	2.3e-10
		Raghusail	HR575925	268	LOC_Os11g35500	Receptor-like protein kinase 5 precursor, putative, expressed	3.7e-18	LRRNT_2	PF08263.5	5.9e-11
		Aijong	HR806748	494	LOC_Os11g35500	Receptor-like protein kinase 5 precursor, putative, expressed	4.1e-29	LRRNT_2	PF08263.5	5.9e-11
		Lal Binni	HR806755	515	LOC_Os04g17940	Retrotransposon protein, putative, unclassified, expressed	6.5e-28	RVT_1	PF00078.20	4.6e-26
		Bhasamanik	HQ832770	457	LOC_Os11g35500	Receptor-like protein kinase 5 precursor, putative, expressed	1.2e-44	LRRNT_2	PF08263.5	5.9e-11
BDTG27	Xa21(A1)	Bangladeshi Patnai	HR806744	366	LOC_Os11g35500	Receptor-like protein kinase 5 precursor, putative, expressed	1.0e-39	LRRNT_2	PF08263.5	5.9e-11
		Raghusail	HR575922	325	LOC_Os11g35500	Receptor-like protein kinase 5 precursor, putative, expressed	2.4e-48	LRRNT_2	PF08263.5	5.9e-11
BDTG28	Xa21(A1)	Bhasamanik	HR806752	359	LOC_Os11g35500	Receptor-like protein kinase 5 precursor, putative, expressed		LRRNT_2	PF08263.5	5.9e-11
BDTG29	Xa21(A1)	Bhasamanik	HR806750	377	LOC_Os11g35500	Receptor-like protein kinase 5 precursor, putative, expressed	1.6e-64	LRRNT_2	PF08263.5	2.6e-10
		IC524526	HR806761	379	LOC_Os11g35500	Receptor-like protein kinase 5 precursor, putative, expressed	2.1e-63	LRRNT_2	PF08263.5	5.9e-11
		Bangladeshi Patnai	HR806745	382	LOC_Os11g35500	Receptor-like protein kinase 5 precursor, putative, expressed	1.4e-62	LRRNT_2	PF08263.5	5.9e-11
		Lal Binni	HR806756	387	LOC_Os11g35500	Receptor-like protein kinase 5 precursor, putative, expressed	1.1e-74	LRRNT_2	PF08263.5	5.9e-11
		IC524502	HR806760	345	LOC_Os11g35500	Receptor-like protein kinase 5 precursor, putative, expressed	1.0e-37	LRRNT_2	PF08263.5	2.6e-10
BDTG30	Xa21(A1)	Gobindobhog	HR806764	323	LOC_Os11g35500	Receptor-like protein kinase 5 precursor, putative, expressed	1.1e-59	LRRNT_2	PF08263.5	5.9e-11
		Bangladeshi Patnai	HR806746	328	LOC_Os11g35500	Receptor-like protein kinase 5 precursor, putative, expressed	1.9e-57	LRRNT_2	PF08263.5	5.9e-11
BDTG31	Xa21(A1)	Bhasamanik	HR806753	376	LOC_Os11g35500	Receptor-like protein kinase 5 precursor, putative, expressed	3.5e-74	LRRNT_2	PF08263.5	5.9e-11
		Lal Binni	HR806758	384	LOC_Os11g35500	Receptor-like protein kinase 5 precursor, putative, expressed	4.8e-72	LRRNT_2	PF08263.5	5.9e-11
BDTG33	Xa21(A1)	Bhasamanik	HR806754	267	LOC_Os11g35500	Receptor-like protein kinase 5 precursor, putative, expressed	1.1e-13			
BDTG34	Xa21(A1)	Raghusail	HR575923	279	LOC_Os11g35500	Receptor-like protein kinase 5 precursor, putative, expressed	2.5e-45	LRRNT_2	PF08263.5	5.9e-11
		Gobindobhog	HR806767	347	LOC_Os11g35500	Receptor-like protein kinase 5 precursor, putative, expressed	3.2e-63	LRRNT_2	PF08263.5	5.9e-11

GenBank Acc No. – accession number of the sequences given by GenBank, L – length of sequence in bp.

### Identification of conserved domains and retrotransposons from the DNA sequences of rare alleles using NCBI and rice genome annotation project database

A total of 23 conserved domains were identified from the 40 rare alleles. The details of homology search and the conserved domain corresponding to the sequence of each rare allele is given in Table 4. Fifteen of the domains were homologous to LRRs. These domains included receptor like kinases (found in 9 sequences), LRR N-terminal domains (found in 4 sequences) and Leucine-Rich Repeats ribonuclease inhibitor (RI)-like subfamily (found in 2 sequences).

The sequences with accession numbers HR575926 and HR575924 (both derived from landrace Raghusail) and HR806763 (derived from landrace IC524526) were homologous to the NB-ARC domain-containing protein having a Pfam hit with BED zinc finger domain (zf-BED). According to Arvind [37] BED-type zinc-finger domain [named after BAEF (boundary element-associated factor) [38] and DREF (DNA replication-related element-binding factor), [39] is found in the *Oryza Xa1* gene. HR614233 and HR575927 were significantly homologous to transcription initiation factor IIA gamma chain, having a Pfam hit with TFIIA_gamma_N. Another sequence HR614234 was homologous to aspartic proteinase nepenthesin-1 precursor having a Pfam hit with Asp or Aspartic proteases family. Sequence JM426580 was significantly homologous to mRNA sequence of the gene *Xa27* of *Oryza sativa* indica. The sequences HR806767 and HR806746 were found to have conserved domains homologous to sugar transferase, and NodB domain of the carbohydrate esterase 4 superfamily.

Conserved domain searches using the Rice Annotation Database revealed the presence of mobile DNA elements within the sequence of 4 of the rare alleles. HQ832768, the sequence of a rare allele from the West landrace Bhasamanik was homologous to an unclassified retrotransposon protein having a Pfam hit of Plant_tran or plant tranposases. The sequence HR806765 from the Mizoram landrace Buhrimtui showed homology with a putative transposon protein, CACTA, En/Spm sub-class of *Oryza sativa* subsp. japonica. According to UniProt database this transposon protein has a molecular function of helicase and hydrolase. JM426578 from the Assam landrace Aijong was significantly homologous to a putative retrotransposon protein of the Ty3-gypsy type. HR806755 from the Assam landrace Lal Binni was significantly homologous to a putative unclassified retrotransposon protein.

## Discussion

The Eastern and North Eastern regions of India are one of the richest reserves of bio-diversity in the country [40]. The inherent variation in the ecotypes of rice, spontaneously evolved in the Eastern State of West Bengal was high enough for scientists to group them as *Oryza sativa* var. benghalensis, at one time [41]. DNA-based markers like SSR and RAPD have been used extensively for the study of such inherent genetic diversity in rice. The results of these studies were also used for unambiguous identification of germplasm and their protection under the trade related intellectual property rights (TRIPS) of the World Trade Organization (WTO). The accessions used in this study were selected from a larger collection to include as much variability as possible based on the agro-morphological data and SSR polymorphism analysis done previously in our laboratory [28,42]. As a follow up of those studies, we aim to extend the search for genetic variability specific to various quality traits and disease resistance abilities. Information on the diversity of disease resistance loci is important to the plant breeders for the identification of diverse donors with major genes and partial resistance. In this preliminary assessment we have tried to find the genetic diversity within six cloned BLB resistance genes in a set of 22 diverse rice accessions using PCR based methods. Even though the sample size is small (22 accessions) it includes accessions of rice varieties from 5 Indian states both aromatic and non-aromatic along with traditional and evolved basmatis and checks.

The PCR profiles of all the 34 primer pairs were clear and consistent. Stutter bands, which were minor products amplified in PCR that has lower intensity than the main allele and normally lacks or has extra repeat units were also present in the profiles of most of the primer pairs [43]. The null alleles were probably due to mutations in the binding region of one or both of the primers, thereby inhibiting primer annealing [30]. The presence of 140 alleles in the 22 accessions indicates high genetic diversity within the 6 BLB resistance gene loci. Analyzing the phenotype-genotype association after actual disease inoculation is requisite for confirming whether the identified rare alleles have any impact on BLB resistance or they are new alleles for BLB resistance. Moreover, the sample size being 22 accessions only, an identified rare allele might no longer be rare after the inclusion of more accessions.

It can be seen from the dendrogram that there was no state-wise or geographical segregation of the accessions based on the obtained polymorphism data. However cluster 1 and 3 consists mostly of the accessions from the North Eastern States. There was some degree of segregation based on whether the accessions were resistant or susceptible. The two resistant landraces from West Bengal, Raghusail and Bhasamanik segregated into a separate major cluster (major cluster A). These two landraces were about 39% similar amongst themselves. The dendrogram also shows instances where susceptible and resistant cultivars have been grouped together. The resistant accessions Kataribhog and IR72 have 89% similarity amongst themselves and they are grouped into cluster 4 along with two susceptible accessions TN1 and Pusa Basmati 1. Another resistant cultivar Bangladeshi patnai is 42% similar to a susceptible, but very popular table rice variety, Dudherswar. Future similar studies incorporating more accessions will confirm whether the alleles generated by the designed primers used here are actually able to segregate accessions on the basis of disease phenotype. Future efforts should concentrate on DNA sequencing, Multiple Sequence alignment and association mapping of all the involved alleles to identify possible linkages between the DNA sequence and the disease phenotype. For improving disease resistance of the aromatic accessions parents may be chosen from major cluster A and B.

According to Zhao et al. [44] most of the knowledge about the genetic architecture of complex traits in rice is based on traditional quantitative trait locus (QTL) linkage mapping using bi-parental populations, which though informative but are not suitable to investigate the genomic potential and tremendous phenotypic variation of the more than 120,000 accessions available in public germplasm repositories. This can only be achieved by documentation of genomic variation at specific loci controlling complex traits using specific genomic region based primers rather than random primers. This variation then has to be coupled with association mapping, a method popularly known as GWA. The information regarding the diversity of domains of the 6 BLB resistant loci obtained in this study is the first step towards such mapping programs. Rather than sequencing all the alleles obtained, only the rare alleles were sequenced in this study. Hence we could not establish any association between the DNA sequence and the resistant and susceptible accessions. For this sequencing of all the alleles and its correlation with disease phenotype are required and these are areas open for future investigation. If such associations can be found, then those will be the forerunner of GWA mapping for BLB resistance loci. In addition to the usual domains like LRR, TFIIA and BED-type zinc-finger, homologies to other conserved domains were also found in this study. The sequence HR806767 was homologous to a sugar transferase domain. Members of sugar transferase family are similar to the pfam00534 Glycosyl transferases group 1 domain. Glycosyltransferases can transfer single or multiple activated sugars to a range of plant molecules, resulting in the glycosylation of plant compounds and plays a key role in in the regulation of plant growth, development and in defense responses to stress environments [45]. Sequence HR806746 is homologous to a Catalytic NodB homology domain of the carbohydrate esterase 4 superfamily. This family catalyzes the N- or O-deacetylation of substrates such as acetylated chitin, peptidoglycan, and acetylated xylan, respectively [46]. The sequence HR614234 is homologous to aspartic proteinase nepenthesin-1 precursor. The *Oryza sativa* constitutive disease resistance 1 (OsCDR1) gene product is an aspartic proteinase that has been implicated in disease resistance signaling. This apoplastic enzyme is a member of the group of ‘atypical’ plant aspartic proteinases [47]. These unusual conserved domains within the rare alleles can be the result of local adaptation. Evaluation of the exact role of these unusual motifs in BLB resistance could be done with the help of disease inoculation and assessment of the disease phenotype. However that was beyond the scope of this study and has been left for future studies.

Transposable elements (TEs) were detected in the DNA sequence of 4 rare alleles. Transposable elements (TEs) are fundamental role players in the variation and adaptive evolution of plant genomes [48-50]. Grass genomes are reported to have active retrotransposons [51]. LTR retrotransposons constitute a major portion of the rice genome [52]. Retrotansposons are activated during stress, wounding and pathogen attack [53,54]. For example transcription of the tobacco retrotransposon Tnt1 could be induced by pathogens and microbial elicitors, as well as by abiotic factors, [55-57]. Moreover Tnt1 insertion could change host gene splicing [58]. A group of LTR retrotransposons was found near the genes encoding the NPR1 disease resistance-activating factor and a heat-shock-factor-(HSF-) like protein in sugarbeet hybrid US H20 [59]. The TEs in this study were found mostly in landraces from the North East or from West Bengal BLB resistant landrace. The probable role of these identified tranaposable elements in this study are yet to be investigated.

## Conclusion

As the name implies, conserved domains of genes are thought to possess little variation. However, this study finds that there is high genetic variability even within the conserved domains of BLB resistance genes in a small set of 22 rice accessions. Environmental stresses including high rainfall, humidity, varied topography and altitude, heavy natural selection pressures of diseases and pests, together with introductions over time and space from adjoining countries like Bhutan, China, Myanmar and Bangladesh; introgression from the wild and weedy relatives, tribal preferences and rituals have been instrumental in the development of this diversity [60]. The inclusion of more genotypes from remote ecological niches and hotspots holds more promise for further allele mining. Future studies should concentrate on DNA sequencing of all the alleles obtained in this study to bring out possible differences between susceptible and resistance accessions. Association mapping after disease inoculation will help to bring out the linkage between the alleles and disease phenotype. Such kind of mapping will be the stepping stone towards genome wide association mapping for BLB resistant loci. Search for transposable elements in the BLB resistance gene loci of the North eastern and resistant rice accessions, and elucidation of their function should form another area of interest.

## Competing interests

The authors declare that they do not have any competing interests.

## Authors’ contributions

BD did all the experiments pertaining to DNA extraction, PCR, PAGE, collected data and was involved in data analysis and drafting of the manuscript. SS procured the rice accessions from various repositories of the North Eastern States, did some of the experimentation pertaining to PCR and PAGE and helped with data collection and analysis and revision of the manuscript. MP did the bootstrap analysis and helped in drafting of the manuscript. TKG was involved with the conception of the work and gave the final approval to the version of the manuscript that is being sent for consideration for publication. All authors read and approved the final manuscript.

## Supplementary Material

Additional file 1: Table S1 Genetic diversity of the six BLB resistant loci in the set of 22 rice accessions. Click here for file
