# Supplementary material for: Genetic diversity of the conserved motifs of six bacterial leaf blight resistance genes in a set of rice landraces
Source: BMC Genet. 2014 Jul 12;15:82. doi: 10.1186/1471-2156-15-82 (PMC4105243; doi:10.1186/1471-2156-15-82)
Supplement: Additional file 1: Table S1 — Genetic diversity of the six BLB resistant loci in the set of 22 rice accessions. [file 1471-2156-15-82-S1.doc]

**Additional file 1: Table S1: Diversity in the six loci in the set of 22 rice accession**

| **Locus** | **Primer name** | **Amplified motif** | **Exon (of the locus)** | **Most variable region of the locus in descending order of variability** | **Number of alleles** | **Rare alleles** | **Null alleles** |
| --- | --- | --- | --- | --- | --- | --- | --- |
| *Xa21(A1)* | BDTG26 | LRR domain | exon1 | 4802bp to 5082bp | 8 | 4 |  |
|  | BDTG29 | LRR domain | exon1 | 5763bp to 6173bp | 8 | 5 | 1 |
| *Xa26* | BDTG18 | receptor kinase domain | exon1, | 4574bp to 5141bp | 8 | 4 | 1 |
| *Xa21* | BDTG20 | Signal domain, | exon 1 | 8bp to 208bp | 7 | - | - |
|  | BDTG21 | LRR domain | exon 2 | 260bp to 760bp | 7 | - | 1 |
|  | BDTG23 | LRR domain | exon 2 | 1279bp to 1880bp | 7 | - | - |
| *Xa27* | BDTG19 |  |  | 1518bp to 1909bp | 6 | 3 | 2 |
| Xa1 | BDTG10 | LRR domain, | exon 4 | 27182bp to 27917bp | 5 | 2 | 6 |
| *Xa21* | BDTG24 | charged domain | exon3 | 1913bp to 2620bp | 5 | 1 |  |
| *Xa21(A1)* | BDTG30 | LRR domain | exon1 | 6140bp to 6531bp | 5 | 2 | 3 |
| *Xa1* | BDTG9 | LRR domain, | exon 4 | 26662bp to 27231bp | 4 | - | - |
|  | BDTG5 | Trans-membrane domain | exon 3 | 5710bp to 6587bp | 4 | - | 2 |
|  | BDTG2 | Kinase 2 and 3 motif | exon 2 | 3602bp to 4031bp | 4 | 1 | 2 |
| *xa5* | BDTG 11 | TFIIA domain | exon 1 | 406048bp to 406306bp | 4 | 1 | 1 |
|  | BDTG 12 | TFIIA domain | exon 2 | 411394bp to 411535bp | 4 | 2 | 1 |
| *Xa26* | BDTG13 | receptor kinase domain | exon1, | 1500bp to 2094bp | 4 | - | - |
|  | BDTG14 | receptor kinase domain | exon1, | 2043bp to 2695bp | 4 | 1 | - |
| *Xa21* | BDTG25 | Kinase domain | exons 4 & 5 | 2651bp to 3919bp | 4 | 1 | - |
| *Xa21(A1)* | BDTG33 | SNAP - O11 transposon | Exon 2 | 7395bp to 7610bp | 4 | 1 | - |
|  | BDTG34 | Kinase domain | exon 3 | 7718bp to 8081bp | 4 | 2 |  |
